# Phenotypes of Motor Deficit and Pain after Experimental Spinal Cord Injury

**DOI:** 10.3390/bioengineering9060262

**Published:** 2022-06-20

**Authors:** Volodymyr Krotov, Volodymyr Medvediev, Ibrahim Abdallah, Arseniy Bozhenko, Mykhailo Tatarchuk, Yevheniia Ishchenko, Leonid Pichkur, Serhii Savosko, Vitaliy Tsymbaliuk, Olga Kopach, Nana Voitenko

**Affiliations:** 1 Department of Sensory Signaling, Bogomoletz Institute of Physiology, 01024 Kyiv, Ukraine; vkrotov@biph.kiev.ua (V.K.); vavo2010@gmail.com (V.M.); arsebo@biph.kiev.ua (A.B.); yevheniia.ishchenko@yale.edu (Y.I.); 2 Department of Neurosurgery, Bogomolets National Medical University, 01601 Kyiv, Ukraine; dr.medyen87@gmail.com (I.A.); tsymb777@gmail.com (V.T.); 3 Department of Restorative Neurosurgery, Romodanov State Institute of Neurosurgery, 04050 Kyiv, Ukraine; mtatarchuk@ukr.net (M.T.); pichkur@neuro.kiev.ua (L.P.); 4 Department of Histology and Embryology, Bogomolets National Medical University, 01601 Kyiv, Ukraine; savosko_s@ukr.net; 5 Department of Clinical and Experimental Epilepsy, Queen Square Institute of Neurology, University College London, London WC1N 3BG, UK; 6 Department of Biomedicine and Neuroscience, Kyiv Academic University, 03142 Kyiv, Ukraine; 7 Research Center, Dobrobut Academy Medical School, 03022 Kyiv, Ukraine

**Keywords:** spinal cord injury (SCI), motor deficit, pain, mechanical allodynia, thermal hypersensitivity

## Abstract

Motor disability is a common outcome of spinal cord injury (SCI). The recovery of motor function after injury depends on the severity of neurotrauma; motor deficit can be reversible, at least partially, due to the innate tissue capability to recover, which, however, deteriorates with age. Pain is often a comorbidity of injury, although its prediction remains poor. It is largely unknown whether pain can attend motor dysfunction. Here, we implemented SCI for modelling severe and moderate neurotrauma and monitored SCI rats for up to 5 months post-injury to determine the profiles of both motor deficit and nociceptive sensitivity. Our data showed that motor dysfunction remained persistent after a moderate SCI in older animals (5-month-old); however, there were two populations among young SCI rats (1 month-old) whose motor deficit either declined or exacerbated even more over 4–5 weeks after identical injury. All young SCI rats displayed changed nociceptive sensitivity in thermal and mechanical modalities. The regression analysis of the changes revealed a population trend with respect to hyper- or hyposensitivity/motor deficit. Together, our data describe the phenotypes of motor deficit and pain, the two severe complications of neurotrauma. Our findings also suggest the predictability of motor dysfunction and pain syndromes following SCI that can be a hallmark for long-term rehabilitation and recovery after injury.

## 1. Introduction

Spinal cord injury (SCI) is a severe medical condition whose annual incidence is estimated to be above 1 million cases [1]. SCI leads to multiple functional impairments that require complex care. Among the most prevalent outcomes of SCI are motor deficits that can exacerbate with time, causing chronic disability and nociceptive hypersensitivity [2,3,4,5,6]. Changes in nociceptive sensitivity can time-dependently reverse back to pre-injury level but more often give rise to chronic pain syndrome, with prevalence rates above 50% [7,8,9,10,11]. Chronic pain, notoriously known for its resistance to treatment, increasingly debilitates an individual’s quality of life and has been classified as a disease in its own right [12]. Despite the fact that motor deficit and chronic pain are common comorbidities after SCI, they have different neuropathological origins. Muscle spasms with hyperreflexia are mediated by the hyperexcitability of motoneurons [13,14], whereas nociceptive hypersensitivity and allodynia arise from changed neuronal excitability and sensory processing by nociceptors [15,16,17]. On the other hand, chronic pain correlates with spasticity [17], suggesting the synergy between these two syndromes after SCI.

Pain developing after injury could be of high variability. A large discrepancy exists between the changes in nociceptive sensitivity reported in either experimental studies or clinical cases—from allodynia (thermal and mechanical modalities) [18,19,20] to a complete loss of sensitivity diagnosed in SCI patients [21,22]. We have also observed a heterogeneous population of SCI animals consisting of rats that experienced intolerable pain, among others [17]. While such pain could be confirmed by self-mutilating behaviour (autotomy) on the injured side, it remained undetectable with classical tactile sensation tests (Hargreaves, plantar von Frey tests). There is a recurrent problem in evaluating tactile sensation in animals with a severe motor deficit of a tested limb [9,10,11]. The implementation of tests that are based on measuring behavioural reactions in response to tactile stimuli of different modalities in animals with heavily restricted motor function arises a common problem that changes in the nociceptive threshold are often underestimated and even are largely compromised due to the animal’s disability to move an injured limb upon testing.

Therefore, in the present study, we assessed motor dysfunction across a relatively large population of injured animals to build up, first, the time courses of motor deficit and, next, to conduct the assessment of nociceptive sensitivity (hyper- versus hyposensitivity) by applying an appropriate nociceptive test for individual animals in accord with their motor function. For an unbiased assessment, we selected a population of animals firmly matched by strain, gender, housing conditions, others and implemented the same evaluation criteria for rating motor deficit between individual animals. We also utilized various SCI models to figure out how the time-dependent recovery of motor function depends on the severity of neurotrauma and the age at the time of injury. Our data show the varied phenotypes of SCI-induced motor deficit, with a mixed pain spectrum (thermal and mechanical modalities), based on several behavioral tests combined with acute H-reflex recordings and post-hoc tissue staining. Our findings shed light on the time courses of motor dysfunction and changes in nociceptive sensitivity based on each animal’s profile for both motor deficit and pain syndromes following neurotrauma of the spinal cord. These data may provide insights for predicting time-dependent rehabilitation and recovery in clinics.

## 2. Materials and Methods

### 2.1. Animals

This study included male Wistar rats. All animal procedures were approved by the local Animal Ethics Committee in Bogomoletz Institute of Physiology and Romodanov Institute of Neurosurgery (Kyiv, Ukraine) and were in full compliance with the ethical guidelines of the European Commission Directive (86/609/EEC) and the International Association for the Study of Pain.

### 2.2. Experimental SCI for Severe Disability: Hemisection with Tissue Excision

The animals were P21–P24 at the time of surgery. For general anesthesia, a mixture of ketamine (70 mg/kg, “Calypsol”, Gedeon Richter, Hungary) and xylazine (15 mg/kg, “Sedazin”, Biowet, Poland) was used (i.p.). The surgery was performed as reported previously in detail [17]. Briefly, after aseptic preparation of the surgical area, one longitudinal incision was made across ~Th8–L2 vertebrae. Laminectomy was carried out at vertebra Th11 or Th12. The left part of the spinal cord was perforated 0.5 mm both rostrally and caudally, as close to the middle vessel as possible, using a 29 G needle; a ~1 mm longitudinal spinal cord incision was made through several transversal cuts by ophthalmic scissors. Then, approximately 1 mm of tissue was removed out of the spinal cord, the opening was covered with subcutaneous fascia, and the wound was sutured. Animals were kept on the heating pad until fully recovered from anesthesia. To avoid post-surgical oedema and inflammation, the animals received bicillin (~600,000 units/kg, Kyiv-med-preparat, Ukraine) and dexamethasone (~4 mg/kg, KRKA, Slovenia) [23].

Sham surgery was performed in the age-matched rats through a similar surgical procedure but without hemisection. The sham group included 15 rats; among those, 1 animal did not recover from anesthesia. The other control group was naïve animals (11 rats). All animals were housed individually.

### 2.3. Experimental SCI for Moderate Motor Dysfunction: Hemisection with Tissue Incision

**Figure 1:**
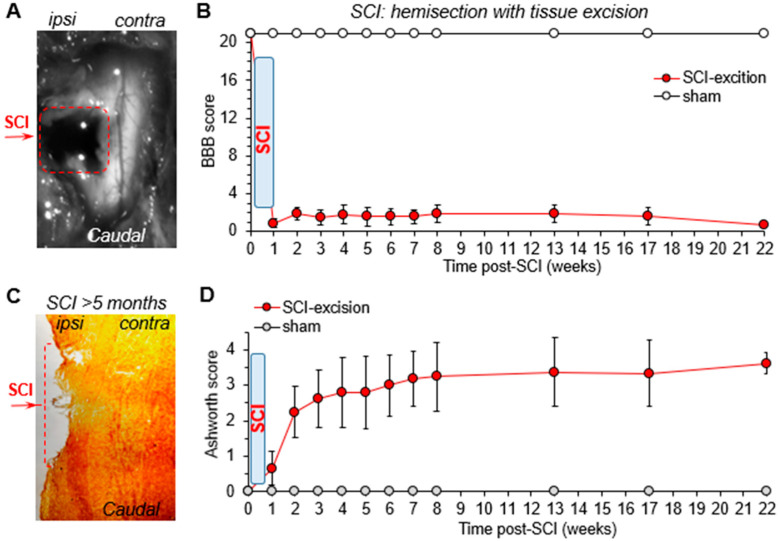
Severe neurotrauma of the spinal cord results in persistent, non-recovered motor disability. (**A**) An image of the spinal cord region (transmitted light) subjected to hemisection with tissue excision. (**B**) The time course of the BBB rating of ipsilateral motor function in experimental groups; animals were ~1 month-old at the time of surgery. (**C**) One example image of a sagittal section of the spinal cord (hematoxylin-eosin staining), showing the hemisection region at 5 months after SCI. (**D**) The time course of Ashworth scaling for muscle tone on the ipsilateral to injury side. Animal groups are the same as for B. Red dashed lines denote the lesion area on both images. Data are mean ± SEM.

**Figure 2:**
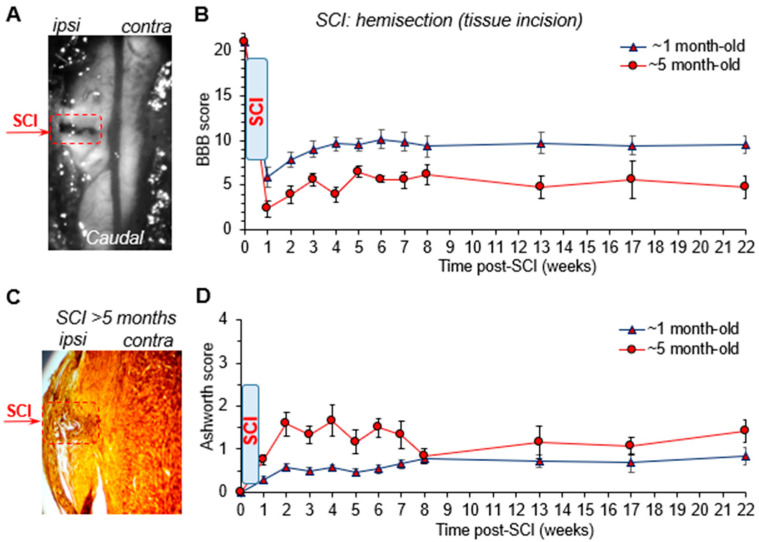
The motor deficit recovery depends on the age at the time of injury. (**A**) A transmitted light image of the spinal cord region, which was subjected to an arguably mild experimental SCI—hemisection with tissue incision. Red dashed line denotes the lesion area. (**B**) The time-course of the motor function on the ipsilateral to injury side (the BBB score) in rats of different ages at the time of injury. (**C**) Image of hematoxylin-eosin staining of the transverse spinal cord tissue at the hemisection region at 5 months after SCI. (**D**) The time-course of muscle tone on the ipsilateral to injury side assessed with the Ashworth scale in young and older SCI rats (same groups as in (**B**)). Data are mean ± SEM.

To model a moderate motor dysfunction, we implemented a hemisection of the spinal cord with tissue incision. The hemisection was performed at the vertebrae ~Th11 (spinal cord segment Th12 or Th13) as we have described above and in the previous study [17], but the left part of the spinal cord was incised transversally starting from the middle vessel (Figure 1A and Figure 2A), using a fine needle and ophthalmic surgical scissors. Two groups of animals were used, different by their age at the time of injury, including P21–P24 (young rats) and ~5-month-old (older animals). Post-operative care of the animals was as described above.

### 2.4. Spinal Cord Tissue Post-Hoc

Post-hoc staining was performed to assess the spinal cord tissue damage for each experimental model used. For this, a block of the tissue at the level of injury was dissected out, fixed in a 10% formalin, and sliced into the approximately 5-mm thick tissue sections, which were placed in formalin. After overnight incubation in increasing concentrations of ethyl alcohol (from 70% to 96%), the tissue sections were replaced in chloroform (for 24 h) and finally in chloroform and paraffin. A standard protocol of hematoxylin-eosin staining was then implemented. Images were acquired using an Axiophot microscope (OPTON, Germany).

### 2.5. Basso, Beattie, Bresnahan (BBB), and Ashworth Scoring of Motor Deficit: The Open-Field Test

All injured animals were rigorously examined postoperatively for the occurrence of motor deficit exclusively on the ipsilateral to injury side. Animals showing a contralateral motor deficit (i.e., the BBB score was below level 10) were immediately discarded, as were the animals that developed a weak ipsilateral motor deficit (i.e., their BBB score was above level 13). The selection of injured animals was typically conducted within one week after post-operational recovery. 

The group of young SCI rats consisted of 40 total animals exposed to hemisection with an incision, in which 2 rats did not recover from anesthesia, and 2 rats revealed neurological deficits on the contralateral side, hence were discarded. The group of older SCI animals (5-month-old) included 9 total rats that were exposed to hemisection with incision, where 2 animals were discarded due to the signs of contralateral deficit and 1 rat displayed the BBB score above level 13 on the ipsilateral to injury side; thus, it was also discarded. The group of young SCI rats with hemisection followed by tissue excision consisted of 11 total rats, 2 displayed the contralateral deficit, and 1 rat had the BBB score >13 on the ipsilateral hind paw; thus, they were discarded. 

Behavioral tests were performed within one week post-surgery and then once a week for at least five months after injury. Both BBB and Ashworth tests were used to assess motor deficit on the ipsilateral to hemisection side, as described in detail earlier [17]. Scoring parameters for both tests were as listed in Table A1 and Table A2. All tests were carried out in a quiet room by the same experimenter for a given test but different experimenters for the motor deficit and pain assessments. 

To quantify the animals’ locomotion and exploratory activity, the open-field test was implemented as detailed previously [24,25]. Briefly, a tested animal was placed in a 75 cm × 75 cm × 40 cm open-field arena and allowed to freely move for a 10-min duration session; the animal’s relocations were recorded with a digital camera (Logitech C270). The total distance travelled was calculated using in-house scripts to quantify the locomotion capability of the animals between different experimental groups.

### 2.6. Nociceptive Threshold of Thermal Modality: The Hargreaves Plantar Test

For the assessment of changes in peripheral sensitivity to thermal (heat) stimulus, the animals were tested using a semi-automated Hargreaves technique, as described in our earlier studies [25,26]. Briefly, a tested animal was allowed to habituate to the experimental chamber (Ugo Basile model 7370 plantar test). Radiant heat was applied to the middle of the plantar surface of one hind paw; the stimulus was automatically turned off once the animal lifted its paw. The time between the stimulus started and the animal lifting its paw was measured, reflecting the nociceptive threshold of thermal modality. From 3 to 5 trials were performed, in a 3 to 5 min interval between each measurement, and the obtained values were averaged. Changes in the thermal nociceptive threshold were also expressed as the maximum possible effect (MPE) by calculating the percentage of the difference between the measured response post-SCI and the baseline latency (control), divided by the difference between the cut-off and the baseline response.

### 2.7. Nociceptive Threshold of Mechanical Modality: Plantar von Frey Test

For the assessment of changes in plantar mechanical sensitivity, the animals were tested using von Frey monofilaments (Bioseb) as described [24]. Briefly, a filament was applied to the plantar surface of one hind paw between the footpads, and the response was considered positive if the animal withdrew the hindlimb upon stimulation. Each filament was applied 10 times in a typically 60-s interval, and the percentage of positive responses was calculated. A larger filament was applied if a tested filament evoked less than 50% of positive responses. The stimulus eliciting 50% of withdrawal responses was the mechanical threshold. The 26-g filament was the cut-off for this test. The plantar test was performed for only the SCI animals able to bear their body weight using all four limbs.

### 2.8. Nociceptive Mechanical Threshold: Dorsal von Frey Test

For assessing changes in mechanical sensitivity of the SCI animals with severe motor dysfunction, which could not support their body weight using four limbs, the dorsal von Frey approach was used as described elsewhere [19,20]. A testing animal was loosely wrapped into a tissue; its hindlimbs were exposed on a horizontal surface while applying a von Frey monofilament to the dorsal surface between the first and the second metatarsal bones. The response was considered positive if animals withdrew the hindlimb upon stimulation; the filaments were applied perpendicularly, arbitrarily starting with the 1-g filament. Each filament was applied 3 times in a typically 60-s interval between trials. The stimulus eliciting two positive responses was the dorsal mechanical threshold. The 60-g filament was the cut-off for this test. 

### 2.9. Nociceptive Mechanical Threshold at the Spinal Level: Spinal von Frey Test 

To examine changes in mechanical sensitivity within the receptive fields at the level of injury, the spinal von Frey approach was implemented [18]. A tested animal was restrained with a towel on a horizontal surface, and a von Frey monofilament was applied to the animal’s back. Testing started with the 4-g monofilament. The response was considered positive if the filament application elicited vocalization. Each filament was applied 10 times in a ~30-s interval between trials. We tested the mechanical nociceptive threshold at the dorsal surface of the thoracic segments (the level of injury) and also at the receptive fields well below.

### 2.10. Hoffman (H)-Reflex

The H-reflex recordings were carried out in all the young SCI animals as an acute experiment, after the termination of behavioral assessment at 5 weeks following injury. For the recordings, the animals were deeply anesthetized, and the sciatic nerve was exposed. A stimulating bipolar hook electrode was placed on the sciatic nerve surroundings while the recording needle electrode was positioned into the gastrocnemius muscle. For nerve stimulation, square pulses of 5-ms duration were applied at 0.2 Hz, starting from 1–2 mA, with increased stimulus intensity (in a 1-mA increment), using a multimodality stimulator system (Neurosoft, Russia). Several trials were recorded (5 sweeps/trial) in at least a 5-min interval. The peak amplitude was measured for both the M- and the H-waveforms, and their ratio was calculated. Measurements were performed in a blind to experimental group fashion.

### 2.11. Statistical Analysis

The data sets are presented as mean ± s.e.m.; n refers to the number of animals tested. A one-way analysis of variance (ANOVA) with Tukey multiple comparisons post-hoc test was used to determine the statistical differences between experimental groups. The Kruskal–Wallis (KW) test with Conover–Iman post-hoc test was used to compare the data sets, which were not normally distributed. The Spearman’s rank correlation coefficient (ρ) was calculated for the parameters of interest. *p* < 0.05 was considered a statistically significant difference between the groups for either test.

## 3. Results

### 3.1. The Time Course of Motor Deficit Depends on the Severity of Neurotrauma 

All injured animals were examined for their motor deficits using the scoring criteria described in Table A1 and Table A2. We evaluated each individual animal before and after injury, starting at week one post-SCI and then for up to five months following injury. This time window was chosen based on the variable periods reported for the recovery of motor function in injured animals across experimental studies [19,27,28]; in some cases, the recovery could take above fourteen weeks [29,30,31]. To establish how the motor dysfunction depends on the severity of neurotrauma, we implemented the following two models of experimental SCI: spinal cord hemisection with tissue excision (a model of severe neurotrauma; Figure 1A,C) or incision (trauma producing arguably mild tissue damage; Figure 2A,C). In the first group of experiments, the animals were young rats (P21–P24 at the time of surgery). The age-matched sham-operated animals were used as the control.

After spinal cord hemisection with tissue excision, each animal developed severe motor disability on the ipsilateral to hemisection side. Soon after the injury, the motor deficit was scored at the lowest BBB rating scale within 1-week post-SCI (mean ± SEM, 0.9 ± 0.5, n = 8; Figure 1A). The disability remained steadily heavy for up to ~5 months; the mean value was 0.8 ± 0.3 at week 22 after injury (Figure 1B). There was no neurological deficit on the contralateral side for the whole observation period (data not shown). The muscle tone—the Ashworth scoring—was on average ~0.7 at week 1 on the ipsilateral hind paw; it further increased with the time after injury (n = 8; Figure 1D). In particular, the mean value raised to ~2.3, 2.6, and 2.8 at weeks 2 to 4, respectively, and exceeded ~3.6 at week 22 post-SCI (n = 8; Figure 1D). All sham-operated animals exhibited no hindlimb dysfunction, neither ipsilateral nor contralateral, at any time tested. The mean values remained constant either for BBB (21, Figure 1B) or Ashworth score (0, Figure 1D). 

Post-hoc staining of the spinal cord (longitudinal tissue sections) has confirmed severe neurotrauma at the level of injury, showing that tissue damage is still there at an extended time period, i.e., ~5 months after SCI. Meanwhile, the hemisection did not cross the corticospinal tract (Figure 1C), ensuring that the observed disability was due to the produced tissue damage.

For modelling less severe neurotrauma, we next implemented a hemisection with tissue incision in young rats (Figure 2A). Indeed, this model produced motor dysfunction that developed to a lesser extent than the model above. At week 1, the injured rats had their average BBB score on the ipsilateral hindlimb of 6.5 ± 1.0 (n = 13, *p* < 0.001 compared with the age-matched animals following hemisection with tissue excision, unpaired *t*-test). Furthermore, the motor deficit gradually recovered (Figure 2B). The BBB score increased to 8.3 ± 0.8 at week 2 and 9.2 ± 0.8 at week 4 (n = 13, *p* < 0.001 compared with the corresponding time point after SCI with tissue excision). Consistent with the BBB, the Ashworth scoring was also significantly lower when compared to the corresponding values in the age-matched rats that were exposed to tissue excision. The Ashworth score value was, on average, ~0.7 vs. ~0.3 at week 1 (*p* < 0.01, unpaired *t*-test), ~2.3 vs. ~0.6 at week 2 (*p* < 0.001), and ~2.8 vs. ~0.6 at week 4 (*p* < 0.01) after a mild injury (tissue incision) vs. severe injury (tissue excision), respectively. The regression analysis revealed a significant trend between the BBB and the Ashworth scores; the Spearman coefficient (ρ) was –0.92 (*p* < 0.001; Figure S1 in Supplementary Materials) at week 4 after SCI.

Post-hoc spinal cord staining has confirmed the tissue damage in this model of a mild motor deficit after 5 months following hemisection with tissue incision (Figure 2C). Although the tissue damage was much lesser than that produced by severe neurotrauma (hemisection with tissue excision), it was still clearly visualized at the level of injury within a similar time window (~5 months after SCI).

Together, these results demonstrate that the profile of injury-induced motor dysfunction depends on the severity of neurotrauma; the recovery of motor deficit, if any, takes place within the first 4 to 5 weeks after injury.

### 3.2. The Recovery of Motor Function Depends on the Age at the Time of Injury 

Thus far, we have established that unlikely severe neurotrauma, a mild spinal cord injury, results in motor dysfunction that showed a time-dependent recovery. To examine whether and how the recovery of motor function depends on the age at the time of injury, we next implemented the established model of a mild injury (hemisection with tissue incision) in older animals. We compared the profile of motor deficit in ~5-month-old rats to that in young rats (~1 month-old at the time of injury).

After hemisection with tissue incision, every older animal developed a severe motor deficit. The BBB score was (mean ± SEM) 2.3 ± 0.9 at week 1 (n = 6, *p* = 0.0266 compared with that in the young group, unpaired *t*-test), 3.9 ± 1.0 at week 2 (*p* = 0.0130) and 3.9 ± 0.8 at week 4 (*p* < 0.001; Figure 2B). The score remained at such a low level for up to 5 months tested (4.8 ± 1.2 at week 22, *p* < 0.01 compared with the corresponding time-point in the young group; Figure 2B). In addition, severe muscle tone developed in each animal of the older group. The Ashworth score was, on average, ~1.6 at week 2 and ~1.7 at week 4 (n = 6, *p* < 0.05 compared with the corresponding time-point in young SCI animals). It remained persistent until the termination of experiments (~5 months after SCI; Figure 2D).

These results clearly demonstrate that the motor function recovery and intrinsic capability of the spinal cord tissue to recover after trauma dramatically deteriorate with age at the time of injury, even in the case of mild neurotrauma.

### 3.3. Young Animals Reveal Two Populations: Those with Persistent or Recovering Motor Deficit after SCI

Our data above have established that motor deficit recovery occurs within the first 4 to 5 weeks after a mild injury in young SCI rats. We aimed next to figure out in detail the profiles of motor dysfunction across all injured animals of the young group within the time window of 4 to 5 weeks post-SCI.

**Figure 3:**
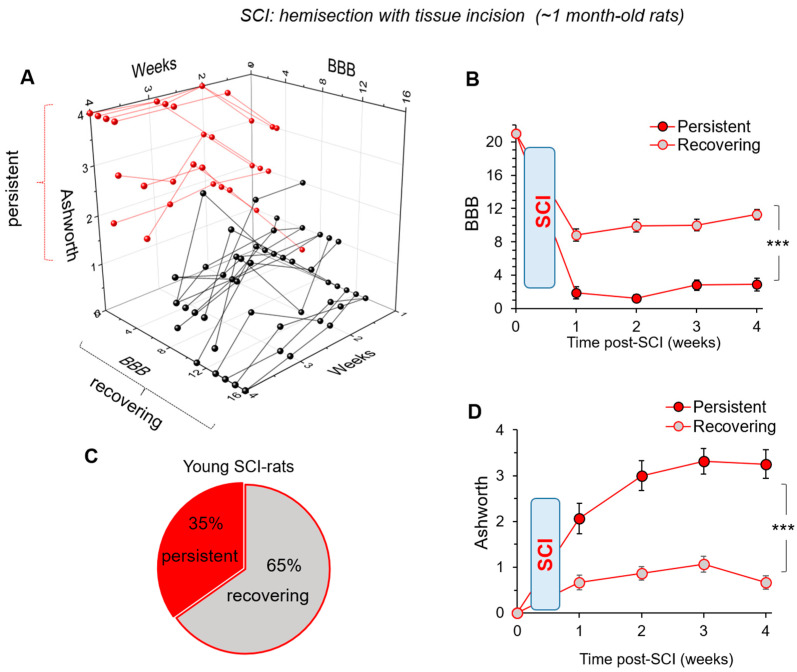
Young SCI rats reveal the following two groups of animals: those with persistent or recovering motor deficits. (**A**) The distribution profiles of changes in the motor function on the ipsilateral to injury side, showing individual BBB scores plotted against the corresponding Ashworth scoring for weeks 1 to 4 after injury. Pooled group of SCI rats, n = 23, consisted of 8 SCI rats with persistent and 15 SCI rats with recovering motor deficit. (**B**) The time course of motor deficit on the ipsilateral to injury side assessed with the BBB score for ‘persistent’ and ‘recovering’ groups of SCI rats. (**C**) The distribution of ‘persistent’ and ‘recovering’ groups of SCI rats based on the time course of motor deficit. (**D**) Same as in (**B**) but for muscle tone assessed with the Ashworth score. Data are mean ± SEM. *** *p* < 0.001 (ANOVA with Tukey post-hoc test).

Similar to the results above, a mild injury (hemisection with tissue incision) produced a motor deficit of the ipsilateral hindlimb, but not the contralateral, in all animals of the young group (n = 23). The BBB score was on average ~6.2, ~6.8, ~7.5, and ~8.3 at weeks 1 to 4, and the Ashworth score was on average ~1.2, ~1.6, ~1.9, and ~1.5 at weeks 1 to 4, respectively (data not shown). The cluster analysis of the distribution profiles across all injured animals revealed that two primary groups appear, in which the scores were principally different, for both BBB and Ashworth, at any time point within the period of interest, 1 to 4 weeks after injury (Figure 3A). One group consisted of animals whose BBB score values were well below 7 at week 1 (8 rats out 23 total), while another group had their scores above 8 (15 rats out 23). In line with the BBB score (Figure 3B), 8 injured rats exhibited severe muscle tone; the Ashworth score was ~2.1 at week 1, while the other 15 animals had a score of ~0.7 (Figure 3D). Notably, the time courses of motor deficit were also principally different between the groups. In a group of 8 SCI rats, the BBB score did not exceed level 7 by week 4, while the other 15 animals recovered up to 11 by week 4 (Figure 3A,B). In accord with the changes in the BBB, 8 SCI rats exhibited severe muscle tone that was even more exacerbated by week 4; the Ashworth value dropped to ~3.3, while there was moderate muscle tone in the other 15 animals between weeks 1 to 4 (Figure 3D). 

Based on these criteria, the animals were categorized into the following two main groups: (i) those that exhibited motor deficit that exacerbates with the time (termed ‘persistent’ thereafter) and (ii) with motor deficit that can attenuate time-dependently (termed ‘recovering’ thereafter). The changes in the BBB score were significant between the ‘recovering’ vs. ‘persistent’ groups for all time points tested (*p* < 0.01, ANOVA; Figure 3B). Furthermore, a recovery trend was significant in the ‘recovering’ group (the slope: 0.76 ± 0.16; t-value: 4.79, *p* < 0.05). The changes in the Ashworth values were also statistically significant between the groups at each time point tested (*p* < 0.001, KW test; Figure 3D). 

In summary, our data revealed that ~35% of young SCI rats exhibited persistent motor deficit (‘persistent’ group), while ~65% displayed motor deficit that time-dependently recovered (‘recovering’ group; Figure 3C).

### 3.4. Changes in the Locomotive Capability after a Mild SCI

**Figure 4:**
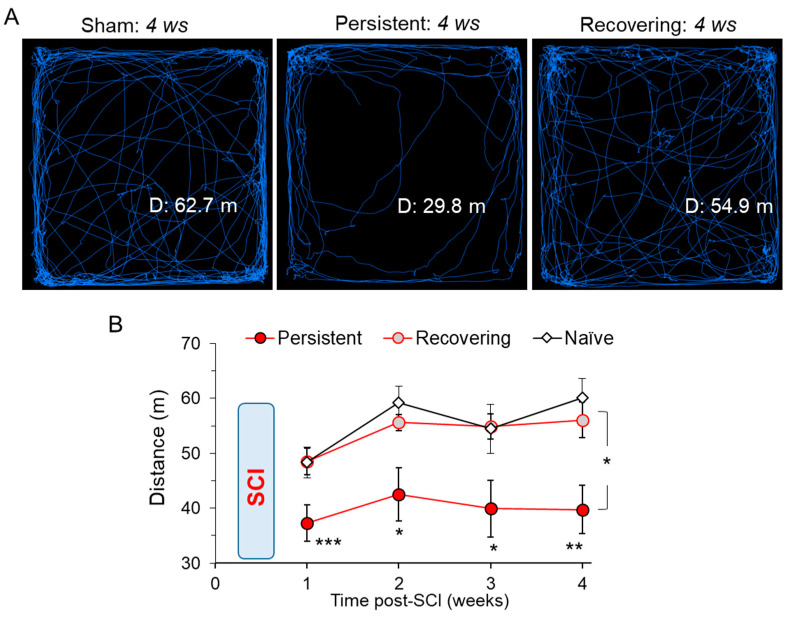
Changes in the locomotive activity between ‘persistent’ and ‘recovering’ groups of inured rats. (**A**) Snapshots show example recordings of the total distance travelled in the open-field arena (10-min duration) by a sham-operated rat and injure rats of the ‘persistent’ and ‘recovering’ groups taken at 4 weeks after injury. (**B**) Statistical summary of the time-dependent changes in locomotive activity in experimental groups. Data are mean ± SEM. * *p* < 0.05, ** *p* < 0.01, *** *p* < 0.001 (ANOVA with Tukey post-hoc test).

Next, we assessed the locomotive activity as a readout of the functional consequence of motor dysfunction produced by mild severity SCI. For quantitative comparisons between the experimental groups, we calculated the total distance travelled across the open-filed arena for each individual animal. The results demonstrated that the animals’ capability for locomotion was substantially different between the ‘persistent’ and ‘recovering’ groups. All animals of the ‘persistent’ group were able to travel little; the total distance travelled was >1.5 times less than that in the control, non-injured littermates at weeks 1 to 4 (*p* < 0.05, ANOVA; Figure 4A,B). Contrary to the ‘persistent’ group, ‘recovering’ SCI animals actively explored their surroundings. For the same recorded time, they could travel, to a similar degree, a distance as their non-injured littermates, naïve or sham-operated animals (n = 15, *p* = 0.1, ANOVA; Figure 4A,B).

No difference was found in locomotive activity between the naïve and sham-operated groups (Figure S2 in Supplementary Materials). The regression analysis demonstrated that locomotive activity was correlated with the motor deficit, either the BBB scores (ρ was 0.61 and 0.45, *p* < 0.05 at weeks 1 and 4, respectively; Figure S3A in Supplementary Materials) or the Ashworth rating scores (ρ was −0.61 and −0.53, *p* < 0.05, respectively; Figure S3B in Supplementary Materials).

### 3.5. Thermal Pain Hypersensitivity after a Mild SCI

**Figure 5:**
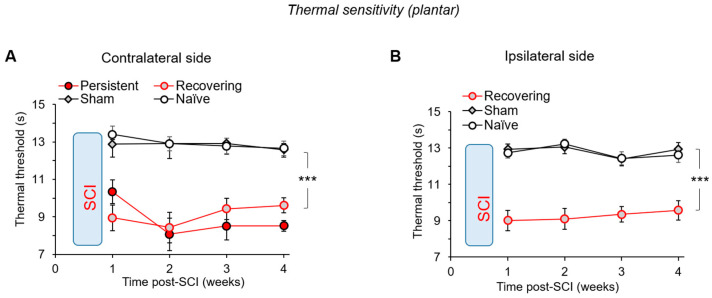
The plantar thermal hypersensitivity after a mild SCI. (**A**) The time-dependent changes in the nociceptive threshold to thermal stimulation—the Hargreaves test latency—show the thermal hypersensitivity on the contralateral hind paw in all injured rats (n = 11 naïve, n = 14 sham-operated animals, n = 8 ‘persistent’ and n = 15 ‘recovering’ SCI animals). (**B**) Same as for A, but for the ipsilateral hind paw. Data are mean ± SEM. *** *p* < 0.001 (ANOVA with Tukey post-hoc test).

We next examined whether and how nociceptive sensitivity is changed in the injured animals in the ‘persistent’ and ‘recovering’ groups. First, we carried out testing of the thermal nociceptive threshold. In full agreement with our previous findings [17], all injured rats developed thermal hypersensitivity on the contralateral hind paw. The hypersensitivity has developed at week 1 (*p* < 0.05, ANOVA) to a similar extent between the two groups of injured animals. The average thresholds were 13.4 ± 0.5 s in the control (n = 11), but 10.3 ± 0.6 s (n = 8) in the ‘persistent’ and 8.9 ± 0.7 s (n = 15) in the ‘recovering’ groups (*p* < 0.01 compared with the naïve or sham-operated groups, ANOVA; Figure 5A). The hypersensitivity of thermal modality persisted over weeks 2 to 4 (*p* < 0.001 compared with the corresponding time-point in naïve or sham-operated animals, ANOVA). There was a significant trend between the thermal hypersensitivity and motor deficit (BBB: ρ = 0.79, *p* < 0.001, Figure S4B in Supplementary Materials; Ashworth: ρ = −0.69, *p* < 0.001; data not shown).

The thermal hypersensitivity was confirmed on the ipsilateral hind paw for the ‘recovering’ SCI animals. A decreased thermal threshold was detected as soon as week 1 (12.7 ± 0.3 s, n = 11 in control to 9.0 ± 0.6 s, n = 15, *p* < 0.001 compared with naïve or sham-operated animals, ANOVA; Figure 5B). Notably, the thermal hypersensitivity showed a trend to recover over weeks 2 to 4 (slope: 0.21 ± 0.02, t-value: 7.94, *p* < 0.05); see also the changes expressed as the MPE (Figure S4A in Supplementary Materials). Similar to the contralateral hypersensitivity, the ipsilateral thermal hypersensitivity correlated with motor deficit (BBB: ρ = 0.72, *p* < 0.001, Figure S4B in Supplementary Materials; Ashworth: ρ = −0.58, *p* < 0.01; data not shown). It was not feasible for the injured animals of the ‘persistent’ group to test the thermal nociceptive threshold on the ipsilateral hind paw due to the animals’ inability to support their body weight with a tested limb.

### 3.6. Changes in the Mechanical Sensitivity: Allodynia vs. Lost Sensation after a Mild SCI

**Figure 6:**
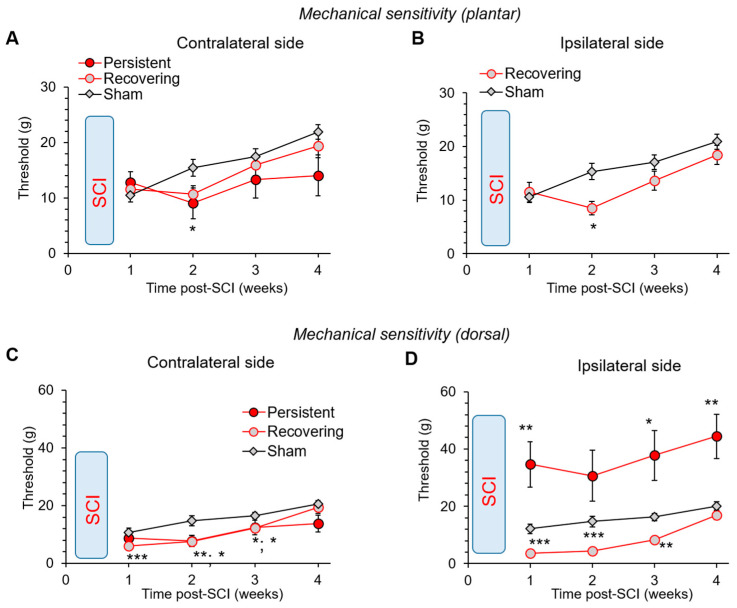
Mechanical allodynia vs. lost sensitivity after a mild SCI. (**A**) The time course of changes in the plantar mechanical threshold on the contralateral hind paw in experimental groups of young rats. (**B**) The time-dependent recovery of the plantar mechanical threshold on the ipsilateral hind paw in injured animals with recovering motor deficit. Note, the SCI rats with persistent motor deficit were not assessed due to animals’ disability to freely move a tested limb. (**C**,**D**) The time course of changes in mechanical threshold on the dorsal surface of the contralateral (**C**) and ipsilateral (**D**) hind paws in experimental groups. All data are mean ± SEM. * *p* < 0.05, ** *p* < 0.01, *** *p* < 0.001 (KW with Conover–Iman post-hoc test).

To examine the changes in mechanical sensitivity after a mild SCI, we started with implementing a classical von Frey filament test, a plantar approach, where appropriate. In line with the revealed hypersensitivity of thermal modality, all injured rats exhibited mechanical hypersensitivity. This was demonstrated as a decreased plantar mechanical threshold on the contralateral hind paw at week 2 post-SCI; 15.4 ± 1.5 g (n = 14) in the sham-operated animals but 9.0 ± 2.8 g (n = 8) in the ‘persistent’ and 10.7 ± 1.5 g (n = 15) in the ‘recovering’ groups (*p* < 0.05 compared with the sham-operated group, KW test; Figure 6A).

Changes in the plantar mechanical threshold on the ipsilateral hind paw were tested for only injured animals of the ‘recovering’ group. We detected a decreased threshold at week 2 (15.3 ± 1.5 g, n = 14 in sham-operated animals vs. 8.5 ± 1.3 g, n = 15 in ‘recovering’ SCI rats, *p* < 0.05, KW test; Figure 6B). This decrease indicates ipsilateral allodynia. It was, however, transient and gradually recovered back to a control level (*p* > 0.05 at weeks 3 and 4 compared with the age-matched sham-operated group, KW test). There was no significant difference in the plantar mechanical threshold between the naïive and sham-operated animals, neither for the ipsilateral (Figure S5A in Supplementary Materials) nor contralateral hind paws (Figure S5B in Supplementary Materials). 

To assess the changes in mechanical sensitivity on the ipsilateral hind paw in the ‘persistent’ group of injured rats, we implemented the dorsal von Frey filament approach. Using this approach, we have detected that all rats of the ‘persistent’ group showed a sharp rise in the ipsilateral mechanical threshold at week 1 (almost 3-fold increase, *p* < 0.01 compared with the age-matched sham-operated animals, KW test; Figure 6D). Such a rise reflects hypoalgesia. This hypoalgesia of mechanical modality progressed by week 4 after injury (Figure 6D). Importantly, none of the tested animals vocalized and/or revealed any attempt to escape the stimulus upon testing. There was also a significant trend between the ipsilateral hypoalgesia and motor deficit over weeks 2 to 4 post-SCI. The Spearman correlation coefficient was –0.76 at week 2 (*p* < 0.05; Figure S6A in Supplementary Materials) and –0.78 at week 4 (*p* < 0.05; Figure S6B in Supplementary Materials). We detected dorsal allodynia on the contralateral hind paw in all injured animals. In the ‘persistent’ group, the contralateral mechanical threshold dropped at week 2 (*p* < 0.05 compared with sham-operated animals, KW test) and remained low until week 4 (Figure 6C). In the ‘recovering’ group, dorsal allodynia was detected on both ipsilateral and contralateral hind paws (*p* < 0.05 for the contralateral and *p* < 0.01 for the ipsilateral sides compared with sham-operated animals, KW test; Figure 6C,D). These data highlight a progressive loss of mechanical sensation on the injured limb of rats with persistent motor deficit, while mechanical allodynia in rats with recovering motor dysfunction.

To assess the changes in mechanical sensitivity at the level of injury (spinal sensation), we applied the spinal von Frey filament approach. We found a drop in the mechanical threshold at week 1, to a similar extent, in all injured animals (Figure S7 in Supplementary Materials). This drop is very likely to be due to the acute effects of laminectomy carried out, as it was also observed in the sham-operated animals. However, such a drop was transient in the sham-operated animals, and their threshold fully recovered back to a control level by week 2; however, it remained lower in all injured animals (*p* < 0.05 compared with the sham group, KW test; Figure S7A in Supplementary Materials). The mechanical allodynia observed at the body trunk remained significant in the ‘persistent’ group by week 4 (*p* < 0.01 compared with the sham group, KW test) but attenuated in the ‘recovering’ animals. Furthermore, we also detected mechanical allodynia at a level well below the injury. This was found in the ‘persistent’ SCI rats at week 2 (*p* < 0.05 compared with the sham-operated or naïve groups, KW test; Figure S7B in Supplementary Materials), but not in the ‘recovering’ SCI rats (*p* > 0.05 compared with sham-operated or naïve animals, KW test; Figure S7B in Supplementary Materials).

### 3.7. H-Reflex Recordings Confirm the Impaired Sensory-Motor Integration after a Mild SCI

Finally, we aimed to validate whether a mild SCI results in impaired sensory-motor integration. For this, we carried out the H-reflex recordings in each young animal tested as an acute experiment after the termination of behavioral assessment. 

**Figure 7:**
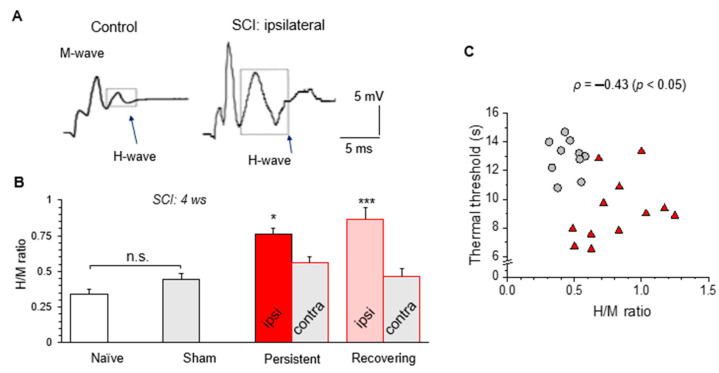
Acute H-reflex recordings show an impaired sensory-motor integration in all injured rats. (**A**) Examples of the H-reflex recorded on the ipsilateral to injury side in control and injured animals at 5 weeks post-operatively. Box denotes the H-wave. (**B**) Summary of the H/M ratio in experimental groups (n = 11 naïve and n = 8 sham-operated animals; n = 18 injured animals those 6 were of the ‘persistent’ group and 12 of the ‘recovering’ group. Data are mean ± SEM. * *p* < 0.05, *** *p* < 0.001 (ANOVA with Tukey post-hoc test). (**C**) The H/M ratio calculated for individual SCI rats was plotted against the corresponding thermal nociceptive threshold. The Spearman correlation coefficient (ρ) is indicated.

In full agreement with our previous study [17], the average ratio of the peak amplitudes between the H- to the M-waves (H/M ratio; Figure 7A) was similar between the naïve (~0.34, n = 11) and sham-operated animals (~0.44, n = 8). However, the H/M ratio significantly increased in all injured rats on the ipsilateral limb. It was, on average, ~0.76 (n = 5) in the ‘persistent’ and ~0.87 (n = 12) in the ‘recovering’ SCI animals (*p* < 0.001 compared with that in either naïve or sham-operated group; Figure 7B). Consistent with the findings above, there was a significant trend between the increased H/M ratio and nociceptive hypersensitivity of thermal modality (ρ = –0.43, *p* < 0.05, Figure 7C). The same trend was found between the increased H/M ratio and motor deficit (BBB: ρ = –0.62, *p* < 0.001; Figure S8 in Supplementary Materials; Ashworth: ρ = 0.52, *p* < 0.01, data not shown).

## 4. Discussion

In the present study, we describe the phenotypes of motor deficit and changed nociceptive sensitivity for different modalities (thermal and mechanical) after experimental SCI. Our regression analysis of the obtained results shows a significant trend between these two severe comorbidities after injury. Our findings further demonstrate how the profile of motor deficit depends on the severity of neurotrauma and the age at the time of injury. Based on the obtained data, a population of injured young rats can be categorized into the following two groups: animals exhibiting persistent motor deficit (~35%) and animals with recovering motor deficit (~65%) after a mild injury. 

Motor deficit following SCI can be of high variability, and a wide range of impairments in motor function was described in the available literature. Such variability could relate to or be, at least partially, mediated by different models of the experimental SCI used, different species (strains) tested, often at different ages, that result in varying severity of the spinal cord tissue damage, hence motor deficit. Among the numerous models of experimental SCI, a lower thoracic hemisection is widely used for modelling unilateral disability [32,33]. The model of unilateral injury at a lower body trunk perfectly suits long-term studies (e.g., in the present study, for half of a year), enabling long-lasting assessment of the injured animals. We also implemented some modifications into a surgical procedure (tissue incision or excision), resulting in severe or mild tissue damage, hence producing a heavy or moderate motor deficit. The motor dysfunction was assessed using the BBB (Table A1) and the Ashworth (Table A2) scores in some experiments for around 5 months, followed by acute H-reflex recordings carried out in each animal tested. Whilst the BBB scoring is routinely used [34,35,36,37], the Ashworth test remains less often implemented in animal studies [38,39], unlike human diagnostics [40,41]. Nevertheless, the Ashworth and the BBB scores in rodents are strongly correlated, based on the earlier [17] and present studies; moreover, a correlation was found between both of the two tests and changed H-reflex. Interestingly, the correlation observed here between the Ashworth scores and changed H-reflex was quite similar to that reported by others in a different injury model [42].

The distribution profiles of motor deficit revealed young injured animals whose motor deficit alleviated within 4 weeks after injury (~65% of the entire cohort) and animals that exhibited persistent motor deficit, comorbid with muscle spasm tone, which even exacerbated with the time after injury (~35%). Our study included a group of 40 young SCI rats, whereas a larger animal cohort would possibly provide a more precise distribution between the groups of injured animals. Notably, the recovery of motor deficit that we observed for the ‘recovering’ group of injured rats occurred within a similar time window (3–4 weeks after injury), as reported elsewhere [18,43]. On the other hand, an occurrence of persistent motor deficit was a primary case for complete spinal cord transection models, with only a few reports for hemisection (see Table A3). Limited recovery of motor function can relate to the injury of reticulospinal and rubrospinal tracts, together with damage to other descending tracts; however, neurological deficits caused by damage to the descending tracts commonly decline within 1–4 weeks [28]. In rats, the reticulospinal tract located in the ventrolateral areas of the white matter of the spinal cord plays a leading control in locomotion; preserving at least 5% of the white matter area of the ventrolateral bundle at the T9–T11 level is required for the restoration of locomotor hindlimb function [28,44,45].

Based on the present data, injured animals with persistent motor deficit represent a substantial group, counting up to 35% of the entire cohort. Such severe motor deficit could not relate to drawbacks in the surgical procedure because every animal was thoroughly evaluated post-operatively, within the first week, to confirm the motor deficit on the injured limb only but also normal motor function on the contralateral side. Immunostaining further confirmed that the lesion did not cross the midline (see also [17]). Finally, the H-reflex recordings, which we carried out in all young animals at the termination of behavioral experiments (5 weeks post-operatively), have proved the impaired sensory-motor integration in all injured animals to a similar extent between the groups. Notably, the observed changes in the H/M ratio were quantitatively similar to those reported by others [46,47]. The prevalent group of young SCI rats consisted of animals whose motor deficit after a mild injury recovered with time. Such innate recovery, although experimentally confirmed, remains enigmatic for its unknown mechanism. Our data demonstrate that a determining role in the recovery of motor function relates to the age at the time of injury. For instance, only young animals (~1-month old) revealed the recovery of motor deficit, opposing a limited recovery in older animals (~5-month-old at the time of injury) subjected to a similar injury. Further studies are required to decipher the precise mechanisms of innate tissue regeneration followed by functional recovery after neurotrauma. 

SCI caused pain of both thermal and mechanical modalities in all injured animals of the young cohort. We found thermal hypersensitivity in all injured rats, using the Hargreaves technique, a semi-automated, less subjective approach to assessing tactile sensation in animals. The bilateral thermal hypersensitivity (on the contralateral and the ipsilateral hind paws) was observed in the animals with recovering motor dysfunction. These changes are in full agreement with our earlier findings [17] and studies by others [18,48,49]. The plantar mechanical hypersensitivity, both ipsilateral and contralateral, was also detected in this group of injured animals; however, it was transient and recovered by week 3 after injury. Similar mechanical hypersensitivity was detected on the contralateral hind paw of animals with persistent motor deficits. In contrast, we also found mechanical hyposensitivity on the injured hind paw of animals with persistent motor deficit (using the dorsal von Frey test). In line with these data, different changes in the animals’ sensitivity after SCI were reported by others [19]. In addition to the changes in the plantar and dorsal mechanical sensitivity, all injured animals displayed spinal mechanical allodynia within the receptive fields at the level of injury and well below. Large interindividual variability in pain patterns has already been reported in different preclinical models of injury-related pain, with attempts to classify nociceptive variability (non-sex-dependent) in rats [50,51,52]. Despite the great scientific interest and a large number of observations of pain heterogeneity between individual animals with similar injury etiology, including changes between the contralateral and ipsilateral sensory abnormalities, the mechanistic basis of such heterogeneity remains unexplored.

Among the possible mechanisms mediating changes in nociceptive sensitivity after SCI, it can be primarily central sensitization of the dorsal spinal cord [17,53] and impairments in the primary nociceptors that, in combination with secondary responses, such as local inflammation [54], ischemic cell death [55], others, may exacerbate pain processing in the sensory pathways. Despite the fact that motor deficit and pain are two separate phenomena and have different neuropathological bases—the hyperexcitability of motoneurons in the ventral spinal cord and sensitization of the superficial dorsal horn, respectively—we have earlier observed chronic pain that correlates with spasticity after SCI [17]. The present study extends this knowledge by describing phenotypes of motor deficit and pain after injury, in addition to a correlation between these complex syndromes. The neurophysiological mechanism(s) mediating a correlation between motor deficit and pain remains enigmatic. Possible interpretations may relate to the earlier described reshuffle between neuronal excitation and inhibition in the dorsal horn, serving as a neuropathological basis for chronic pain hypersensitivity and allodynia in animals with persistent motor deficit. The loss of ipsilateral mechanical sensitivity in these animals is caused by paralysis after damage to half of the spinal cord and lost proprioception (Brown-Sequard’s hemiplegia). Some studies suggest functional connectivity between the dorsal and ventral horns [56,57], which disruption was visualized with magnetic resonance imaging [58]. Spontaneous axonal sprouting post-SCI [59,60] may, hypothetically, contribute to the motor deficit directly via supraspinal inhibition of motoneurons and indirectly by inhibiting the sensory-evoked depolarization of motoneurons—the synergistic effect that requires functional connectivity. The hyperexcitability of the dorsal horn and the ventral horn might be linked via interconnections between the dorsal horn interneurons with interneurons of the deeper laminae. For instance, the laminae IV–V interneurons were found projecting ipsilaterally to motoneurons of the ventral horn [61]. In support of this, the hyperexcitability of motoneurons could be reduced by targeting sensory transmission to motoneurons [62]. Although the present study provides evidence for a trend between motor deficit and pain after neurotrauma, future studies are required to highlight the mechanistic basis for such phenomena at the cellular level.

## 5. Conclusions

Our data describe the phenotypes of motor deficit and pain after experimental SCI and show how the recovery of motor disability depends on the severity of neurotrauma and the age at the time of injury. Our findings also demonstrate a trend between motor deficit and pain and identified distinct groups based on each animal’s time course for motor dysfunction and sensory abnormalities in animals subjected to identical injury. Although this study does not explain the mechanistic basis behind the identified heterogeneity and the revealed correlation, it provides a platform for understanding the profiles of motor deficit and pain, two chronic comorbidities of SCI. This new knowledge may serve as a prognosis on the recovery following injury and, thus, help manage timely treatment.

## Data Availability

All data generated and/or analyzed during this study are included in this article and its Supplementary Material files.

## Supplementary Materials

The following supporting information can be downloaded at: https://www.mdpi.com/article/10.3390/bioengineering9060262/s1, Figure S1: Regression analysis between the BBB and the Ashworth score values measured in individual rats after experimental SCI of mild severity; Figure S2: Locomotive activity in the groups of sham and naïve animals does not change with time; Figure S3: Changes in the locomotive activity correlate with the motor deficit after a mild SCI; Figure S4: The thermal hypersensitivity correlates with the motor deficit after a mild SCI; Figure S5: Plantar mechanical sensitivity in naïve and sham-operated groups of young animals; Figure S6: Regression analysis between the motor dysfunction and the mechanical sensitivity on the injured hind paw after a mild SCI; Figure S7. Changes in mechanical sensitivity at the spinal level (spinal surface assessment) after a mild SCI; Figure S8: Regression analysis between the increased H/M ratio and the motor deficit in injured animals.

## Appendix A

**Table A1:** Scoring for the BBB rating scale in rats after unilateral SCI.

Score	Criteria
0	No hind limb movement
1	Slight (less than half movement) of one or two joints (the hip and/or the knee)
2	Extensive movement of one joint (more than half of the normal range)
3	Extensive movement of two joints
4	Slight movement of all three joints
5	Slight movement of two joints and extensive movement of the third
6	Extensive movement of two joints and slight movement of the third
7	Extensive or full movement of all joints
8	Sweeping with no weight support or plantar placement of the paw with no weight support
9	Weight support with plantar placement of the paw with stationary position or occasional, frequent, or consistent weight supported dorsal stepping and no plantar stepping
10	Occasional weight supported plantar steps
11	Frequent to consistent weight supported plantar steps
12	Frequent to consistent weight supported plantar steps and occasional coordination between fore and hind limbs
13	Frequent to consistent weight supported plantar steps and frequent between fore and hind limbs
14	Consistent weight supported plantar steps, consistent coordination
15	Consistent plantar stepping and consistent coordination; and no toe clearance or occasional toe clearance during forward limb advancement; predominant paw position is parallel to the body at initial contact
16	Consistent plantar stepping and consistent coordination during gait; and toe clearance occurs frequently during forward limb advancement; predominant paw position is rotated when raising and parallel at contact with the surface
17	Parallel positioning of the paw relative to the rostrocaudal axis when raising and at contact with the surface

**Table A2:** Scoring for the Ashworth rating scale in rats after unilateral SCI.

Score	Criteria
0	No increase in muscle tone
1	Slight increase in tone when limb is moved in flexion or extension
2	More marked increase in tone, with limb still easily flexed
3	Considerable strength of tone with difficult passive movement
4	Limb rigid in flexion or extension

**Table A3:** The BBB rating scores in different SCI models across various studies.

BBB Score	Weight/Age	Model	Reference
18	200–250 g	Hemisection	[18]
15–17	100–125 g	Hemisection	[19]
15	250–320 g	Hemisection	[36]
10–15	165–550 g	Hemisection	[43]
11–13	210 g	Hemisection	[46]
12–14	8 weeks	Hemisection	[29]
12	220–250 g	Hemisection	[63]
6–10	8 weeks	Hemisection	(Figure 2A)
2–5	5 months	Hemisection	(Figure 2A)
4–5	200–220 g	Hemisection with excision	[30]
4–5	180–200 g	Hemisection with excision	[31]
1–3	8 weeks	Hemisection with excision	(Figure 1A)
3–7	260–280 g	Transection	[64]
3	225–250 g	Transection	[65]
