# Supplementary material for: Phenotypes of Motor Deficit and Pain after Experimental Spinal Cord Injury
Source: Bioengineering (Basel). 2022 Jun 20;9(6):262. doi: 10.3390/bioengineering9060262 (PMC9220047; doi:10.3390/bioengineering9060262)
Supplement: Supplementary file 1 [file bioengineering-09-00262-s001.zip › bioengineering-1728237-supplementary.pdf]

bioengineering

Supplementary Materials

Phenotypes of Motor Deficit and Pain after Experimental Spinal Cord Injury

Volodymyr Krotov [1,†], Volodymyr Medvediev [1,2,†], Ibrahim Abdallah [2], Arseniy Bozhenko [1], Mykhailo Tatarchuk [3], Yevheniia Ishchenko [1,‡], Leonid Pichkur [3], Serhii Savosko [4], Vitaliy Tsymbaliuk [2,3], Olga Kopach [1,5,*] and Nana Voitenko [1,6,7,*]

[1] Department of Sensory Signaling, Bogomoletz Institute of Physiology, 01024 Kyiv, Ukraine; vkrotov@biph.kiev.ua (V.K.); vavo2010@gmail.com (V.M.); arsebo@biph.kiev.ua (A.B.); yevheniia.ishchenko@yale.edu (Y.I.)
[2] Department of Neurosurgery, Bogomolets National Medical University, 01601 Kyiv, Ukraine; dr.medyen87@gmail.com (I.A.); tsymb777@gmail.com (V.T.)
[3] Department of Restorative Neurosurgery, Romodanov State Institute of Neurosurgery, 04050 Kyiv, Ukraine; mtatarchuk@ukr.net (M.T.); pichkur@neuro.kiev.ua (L.P.)
[4] Department of Histology and Embryology, Bogomolets National Medical University, 01601 Kyiv, Ukraine; savosko_s@ukr.net
[5] Department of Clinical and Experimental Epilepsy, Queen Square Institute of Neurology, University College London, London WC1N 3BG, UK
[6] Department of Biomedicine and Neuroscience, Kyiv Academic University, 03142 Kyiv, Ukraine
[7] Research Center, Dobrobut Academy Medical School, 03022 Kyiv, Ukraine
* Correspondence: o.kopach@ucl.ac.uk (O.K.); nana@biph.kiev.ua (N.V.)
† These authors contributed equally to this work.
‡ Present address: Department of Molecular Biophysics and Biochemistry, Yale University, New Haven, CT 06520, USA.

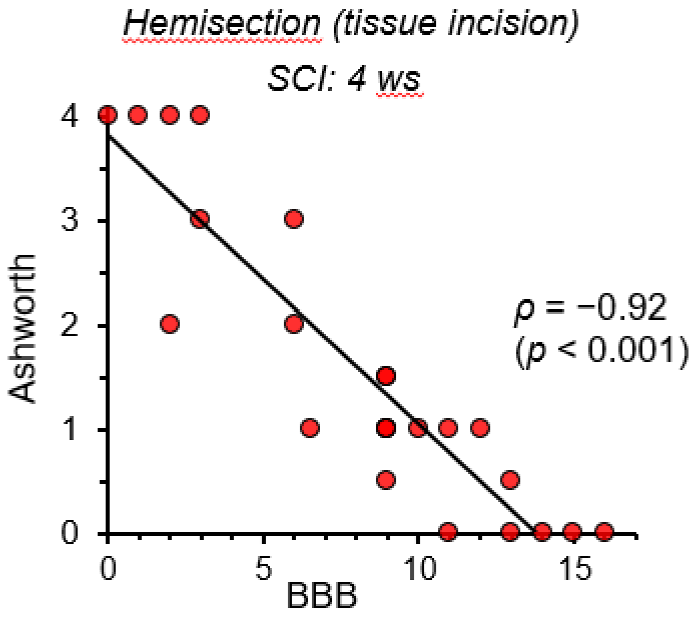

Figure S1. Regression analysis between the BBB and the Ashworth score values measured in individual rats after experimental SCI of mild severity. The BBB score values are plotted against the corresponding Ashworth score measured in individual animals of the young group (~1 month-old) at week 4 after a mild injury (hemisection with tissue incision). The Spearman correlation coefficient (ϱ) indicated.

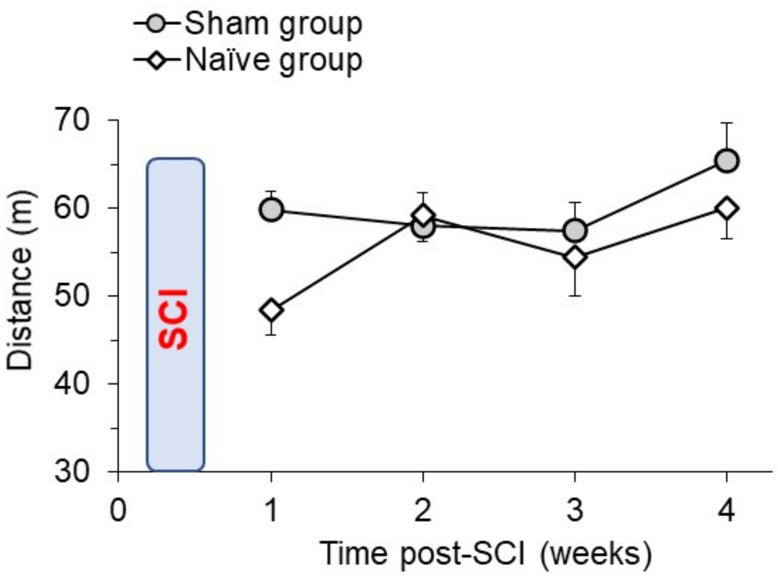

Figure S2. Locomotive activity in the groups of sham and naïve animals does not change with time. The time-course of changes in animal locomotor activity, analysed as the total distance travelled in the open-field arena (a 10-min duration of the test), in the sham-operated and naïve animals. Data are mean ± SEM.

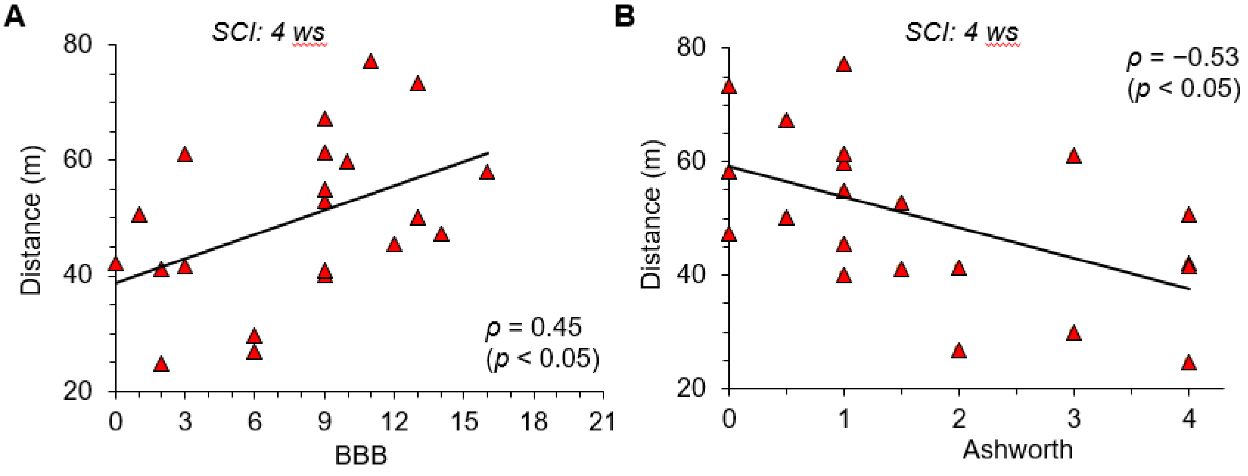

Figure S3. Changes in locomotive activity correlate with the motor deficit after a mild SCI. The total distance travelled by individual SCI animals plotted against the corresponding BBB (**A**) and Ashworth scores (**B**). The Spearman correlation coefficient (ϱ) is indicated (n = 23 injured rats).

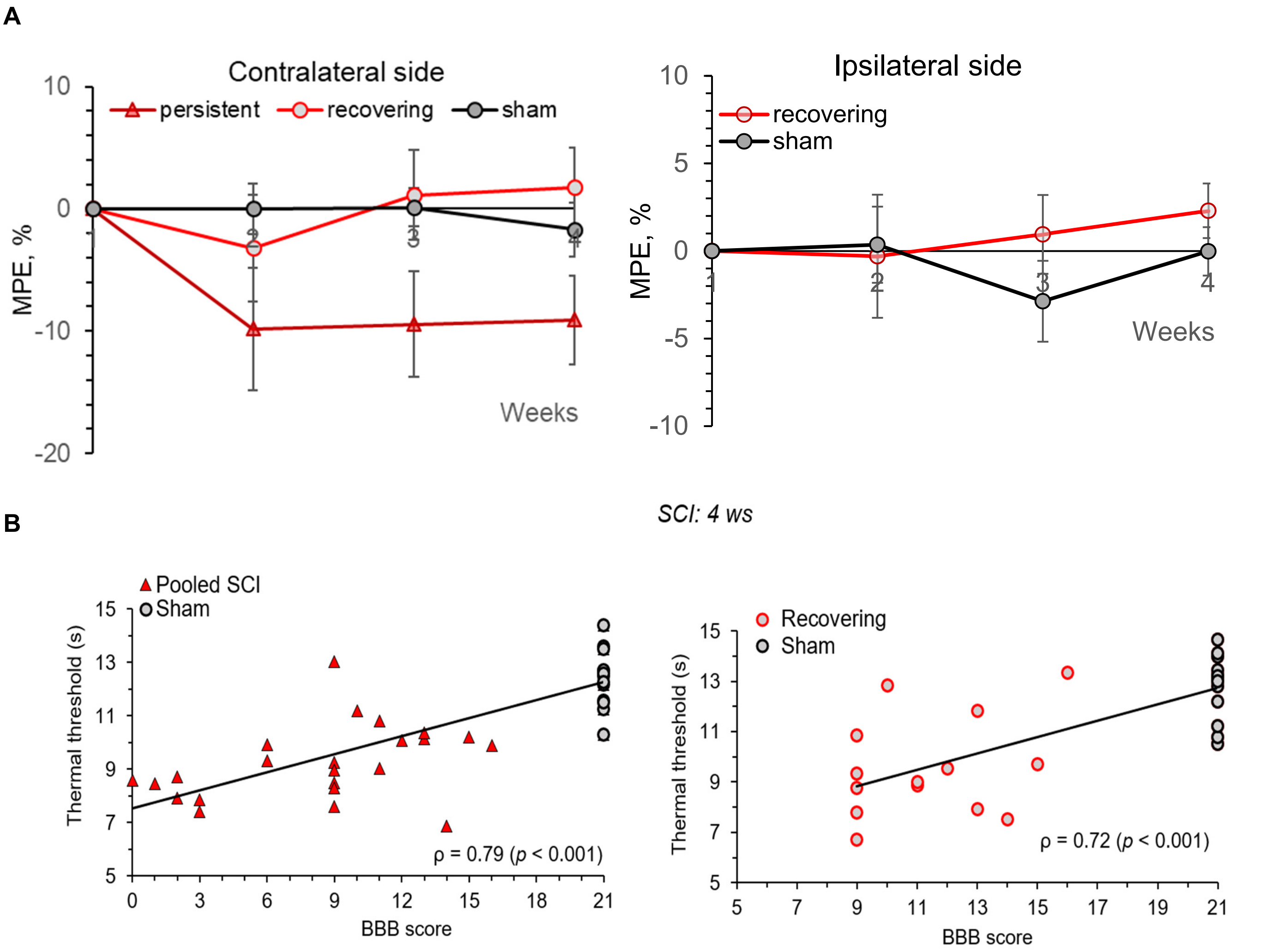

Figure S4. The thermal hypersensitivity correlates with the motor deficit after a mild SCI. **(A)** The time course of maximum possible effect (MPE) of thermal nociception on the contralateral (left) and ipsilateral (right) hind paws in experimental groups. **(B)** The thermal nociceptive threshold of individual SCI animals plotted against the corresponding BBB in the pooled group of SCI rats (left) and the recovery group (right) at 4 weeks post-SCI. The Spearman correlation coefficient (ϱ) indicated.

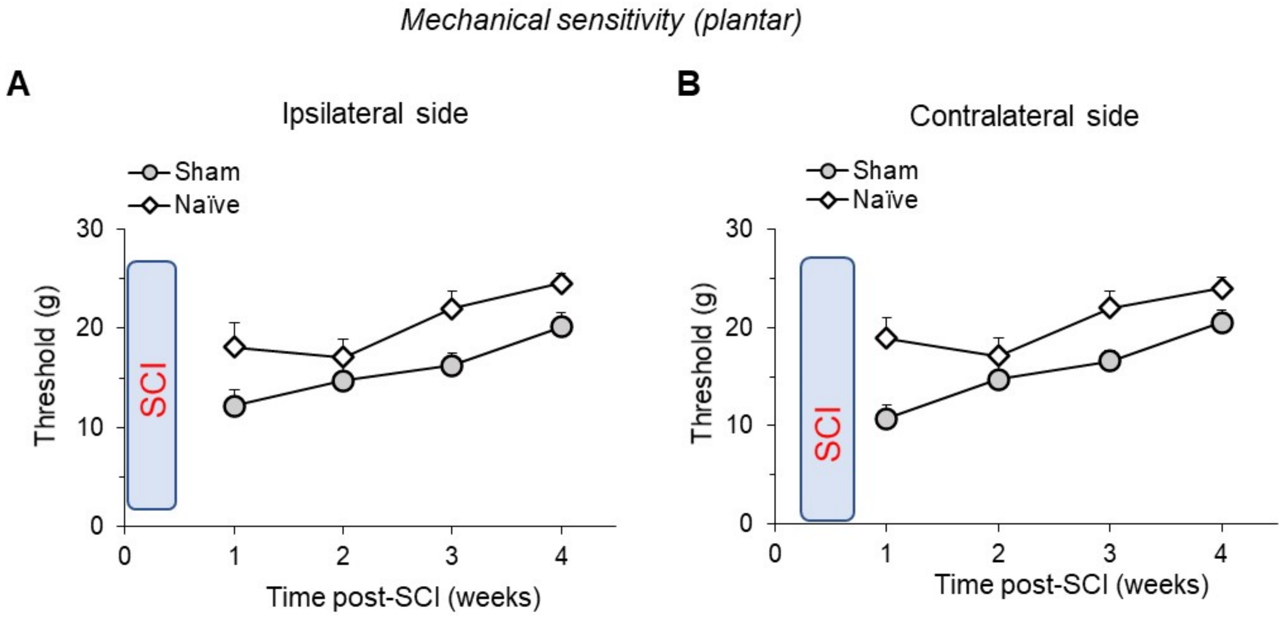

Figure S5. Plantar mechanical sensitivity in naïve and sham-operated groups of young animals. The time course of changes in the plantar mechanical sensitivity on the ipsilateral (**A**) and contralateral hindpaws (**B**) in control groups of young animals. All data are mean ± SEM.

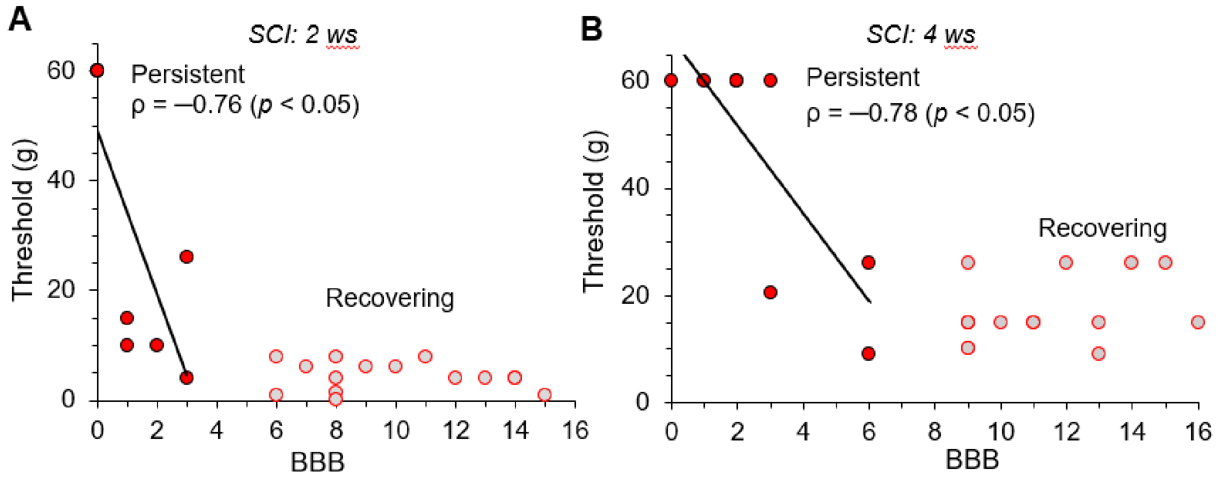

Figure S6. Regression analysis between the motor dysfunction and the mechanical sensitivity on the injured hindpaw after a mild SCI. The mechanical threshold measured on the dorsal surface of the ipsilateral hindpaw in individual animals plotted against the corresponding BBB score for each of the injured rats, in the 'persistent' and 'recovering' groups, at week 2 (**A**) and week 4 (**B**) after experimental SCI. The Spearman correlation coefficient (ϱ) is indicated for the group of SCI-rats with persistent motor deficit.

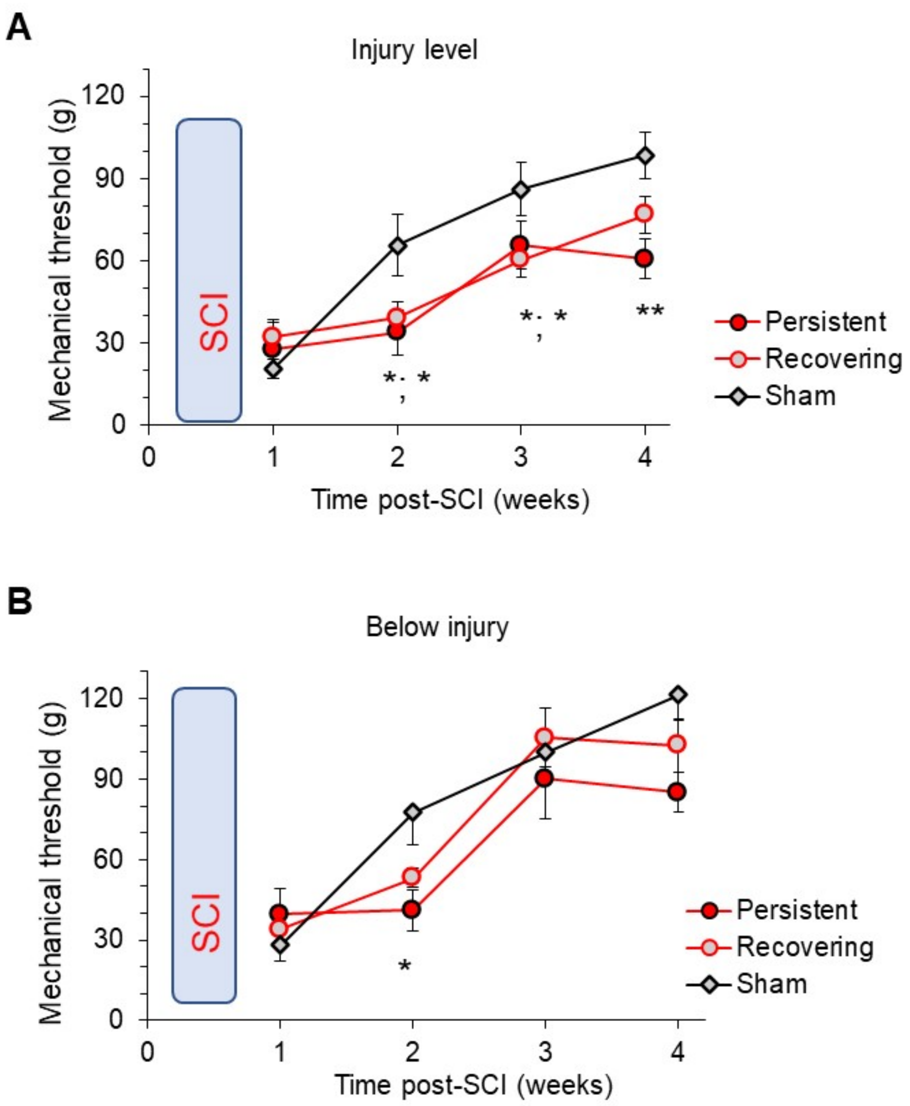

Figure S7. Changes in mechanical sensitivity at the spinal level (spinal surface assessment) after a mild SCI. The time course of changes in the mechanical threshold at the level of injury (**A**) and well below (**B**) in different experimental groups. All data are mean ± SEM. **P* <0.05, ***p* <0.01, ****p* <0.001 (KW with Conover-Iman post-hoc test).

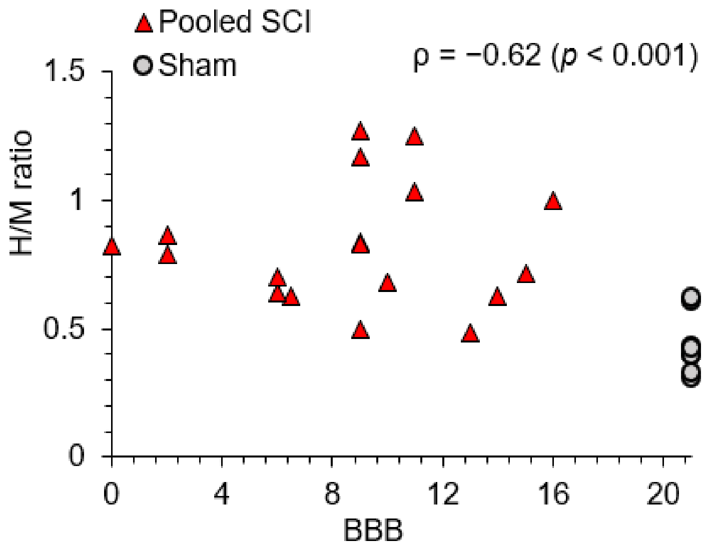

Figure S8. Regression analysis between the increased H/M ratio and the motor deficit in injured animals. The H/M ratio calculated for individual animals plotted against the corresponding BBB score. The Spearman correlation coefficient (ϱ) indicated.
